# Comparison of intravenous efgartigimod and intravenous immunoglobulin in patients with Guillain–Barré syndrome

**DOI:** 10.1186/s13023-025-04060-0

**Published:** 2025-10-21

**Authors:** Huiqiu Zhang, Feipeng Zhai, Menghan Su, Yi Zhang, Jing Ma, Junsen Zhao, Juan Wang, Xueli Chang, Yi Liu, Junhong Guo, Wei Zhang

**Affiliations:** 1 Department of Neurology, First Hospital, Shanxi Medical University, No. 85, Jiefang South Street, Taiyuan, 030012 China; 2 Research Center for Neurological Diseases, Shanxi Medical University, Taiyuan, China; 3 Department of Neurology, Shanxi Provincial People’s Hospital, Taiyuan, China; 4 First Clinical Medical College, Shanxi Medical University, Taiyuan, China

**Keywords:** Guillain–Barré syndrome, Efgartigimod, IVIg, Neurofilament light chain, Anti-GM1 antibody

## Abstract

**Objective:**

This study aimed to compare the effectiveness of intravenous efgartigimod and intravenous immunoglobulin (IVIg) in patients with Guillain–Barré syndrome (GBS).

**Methods:**

This dual-center, retrospective study analyzed prospectively collected data from adult patients with severe GBS who received either efgartigimod or IVIg. The primary outcome was the proportion of patients who achieving a GBS Disability Scale (GBS-DS) score ≤ 2 at 4 weeks post-treatment. Secondary outcomes included the proportion of patients achieving GBS-DS ≤ 2 at week 24; ≥ 1-grade improvement in GBS-DS at weeks 4 and 24; GBS-DS grade at week 4; and changes in GBS-DS, Medical Research Council (MRC) sum score, and other validated disability measures at weeks 1, 2, 4, 8, 16, and 24. Baseline serum levels of neurofilament light chain (NfL) and anti-GM1 antibodies, and their dynamic changes at 1 week post-treatment were assessed as exploratory outcomes.

**Results:**

Twenty-one patients were enrolled (efgartigimod: *n* = 9; IVIg: *n* = 12). The primary outcome was not achieved (OR = 0.67, 95% CI [0.10, 4.48]; *P* = 1.000). Although most secondary outcomes did not reach statistical significance, the MRC sum score demonstrated significantly greater improvement in the efgartigimod cohort than in the IVIg cohort (*P* = 0.007). In addition, efgartigimod demonstrated significantly more favorable trajectories of NfL levels and anti-GM1 antibody titers compared with IVIg (*P* < 0.001).

**Interpretation:**

Efgartigimod demonstrated superiority in one secondary outcome and two exploratory measures, suggesting its potential as alternative to IVIg in GBS management.

**Supplementary Information:**

The online version contains supplementary material available at 10.1186/s13023-025-04060-0.

## Introduction

Guillain–Barré syndrome (GBS), the most common cause of acute flaccid paralysis, is an immune-mediated polyradiculoneuropathy [1, 2]. It typically manifests as rapidly progressive limb weakness and can lead to respiratory insufficiency within hours to days, sometimes resulting in death [2, 3]. The main subtypes of GBS include acute inflammatory demyelinating polyradiculoneuropathy (AIDP), acute motor axonal neuropathy (AMAN), and acute motor sensory axonal neuropathy (AMSAN) [4]. Immunoglobulin G (IgG) autoantibodies play a key role in GBS pathogenesis, with molecular mimicry being the mechanism by which infectious agents trigger an immune response against autoantigens [5–7].

Intravenous immunoglobulin (IVIg) and plasma exchange (PLEX) are regarded as first-line therapies for GBS and offer substantial benefit [8]. However, despite the application of these approaches, approximately 3% to 7% of patients die, and 20%-30% require mechanical ventilation [9]. In addition, IVIg and PLEX are not always available [10], especially given the growing global shortage of blood products. As a result, there is an urgent need to develop effective and safe therapeutic drugs for GBS that are independent of blood-derived products and suitable for industrial-scale production.

Efgartigimod, a human IgG1-derived Fc fragment, acts as a high-affinity ligand for the neonatal Fc receptor (FcRn), promoting accelerated IgG degradation and achieving rapid IgG level reduction with an acceptable safety profile [11]. Its clinical efficacy has been validated in multiple IgG autoantibody-driven disorders, including myasthenia gravis (MG) and chronic inflammatory demyelinating polyneuropathy (CIDP) [12–16]. We previously reported the first two GBS patients treated with intravenous efgartigimod monotherapy, both of whom showed clinically meaningful improvements in functional outcomes alongside a favorable safety profile [17]. Importantly, no head-to-head trials have systematically compared efgartigimod with IVIg in GBS. This retrospective dual-center cohort study provides the first comparative analysis of therapeutic response and safety outcomes between these two immunomodulatory treatments.

## Methods

### Study design and participants

This dual-center retrospective study analyzed prospectively collected data from the First Hospital of Shanxi Medical University and Shanxi Provincial People’s Hospital between November 2023 and March 2025. Patients with GBS who received either intravenous efgartigimod or IVIg were included in the efgartigimod and IVIg cohorts, respectively. The choice of treatment was based on the availability of IVIg at the time of admission, as well as patient preference. Eligible patients met all inclusion criteria: a confirmed diagnosis of GBS according to the criteria of the National Institute of Neurological Disorders and Stroke (NINDS) and the Brighton Collaboration [8, 18]; age ≥ 18 years; onset of weakness within 2 weeks prior to treatment; inability to walk unaided for ≥ 5 m; receiving IVIg (400 mg/kg over 5 days) or intravenous efgartigimod (10 mg/kg, twice within 5 days) treatment; provision of signed informed consent; and a minimum follow-up of 4 weeks. The exclusion criteria included undergoing PLEX therapy; recent use of immunosuppressive (e.g., azathioprine, cyclosporine, tacrolimus, or prednisolone daily within 4 weeks); receipt of rituximab within 24 weeks; active malignancies with uncontrolled progression; severe cardiopulmonary comorbidities; or combined IVIg-efgartigimod therapy. This dose of efgartigimod was selected based on Phase I PK/PD data showing near-maximal IgG reduction at 10 mg/kg, whereas higher doses did not further enhance IgG lowering but increased the risk of adverse events [11]. Twice were administered within the first five days as pathogenic antibody titers in GBS typically peak during the first week [19]. Additionally, age- and sex-matched healthy controls (HCs) were recruited from the Health Examination Center of the First Hospital of Shanxi Medical University to serve as a comparator cohort for exploratory biomarker analyses.

This study has been approved by the Institutional Ethics Committee of the First Hospital of Shanxi Medical University (KYLL-2025-090) and performed in accordance with the ethical standards laid down in the 1964 Declaration of Helsinki and its later amendments. Clinical trial number: not applicable. All participants provided written informed consent.

### Data collection and clinical evaluation

Demographic data, antecedent events (such as diarrhea, respiratory infections, or fever), nerve conduction studies (NCS) and laboratory tests, including serum albumin (reference range 40–55 g/L) and serum IgG levels (reference range 8.6–17.4 g/L) were collected. NCS results were classified as demyelinating, axonal, or other types based on the Hadden criteria [20]. According to clinical and NCS data, patients were classified into AIDP, AMAN, AMSAN or unclassified as described previously [21].

The GBS Disability Scale (GBS-DS) [22], Inflammatory Neuropathy Cause and Treatment (INCAT) disability score [23], Inflammatory Rasch-Built Overall Disability Scale (I-RODS) [24], grip strength (assessed via an electronic hand dynamometer, CAMRY, Model EH101, China), and Medical Research Council (MRC) sum score [25] were evaluated at baseline and at weeks 1, 2, 4, 8, 16, and 24 post-treatment. Follow-up duration was calculated from the date of initial efgartigimod or IVIg administration.

### ELISA

Serum samples were prospectively collected at baseline and 1-week post-administration, followed by centrifugation at 1,500 × g for 10 min at 4 °C. The supernatants were immediately frozen and stored at -80 °C until assayed. Enzyme-linked immunosorbent assay (ELISA) kits (Jiangsu Meimian Industrial Co., Ltd) were used to quantify neurofilament light chain (NfL) levels and detect the IgG antibody against GM1 following the manufacturer’s instructions.

### Outcomes

Study outcomes were specified before data analysis. The primary outcome was defined as the proportion of patients attaining GBS-DS functional grade ≤ 2 at 4 weeks post-treatment. Secondary outcomes included: (1) proportion of patients with ≥ 1-grade improvement in GBS-DS from baseline at weeks 4 and 24; (2) proportion of patients with GBS-DS ≤ 2 at week 24; (3) GBS-DS grade at week 4; and (4) mean changes from baseline in GBS-DS, INCAT disability score, I-RODS, grip strength, and MRC sum score at prespecified timepoints (weeks 1, 2, 4, 8, 16, and 24). Serum NfL and anti-GM1 level trajectories were pre-defined as exploratory outcome measures to evaluate axonal injury dynamics in this retrospective cohort.

Adverse events and serious adverse events were recorded within 1 month after the initial dose and graded according to the Common Terminology Criteria for Adverse Events (CTCAE) Version 5.0 [26]. Disease progression or events definitely related to disease were not considered adverse events, as reported in previous studies [27, 28].

### Statistical analysis

Statistical analyses were performed using GraphPad Prism 8 (GraphPad Prism, Inc., San Diego, CA, USA) and SPSS version 27 (SPSS Inc., Chicago, IL, USA). Continuous variables with normal distribution were expressed as the mean ± standard deviation (SD), whereas nonnormally distributed data were presented as the median with interquartile range (IQR). The Shapiro–Wilk test was used to assess normality, and Levene’s test was applied to evaluate homogeneity of variance. Baseline intergroup comparisons were performed via independent-samples *t* tests for parametric data or Mann‒Whitney *U* tests for nonparametric data. Categorical variables are presented as frequencies and percentages, with group differences evaluated using Fisher’s exact test. Repeated measures during follow-up were analyzed using a generalized linear mixed model (GLMM), adjusting for time and accounting for within-patient correlation. Sensitivity analyses were performed for both the primary and secondary outcomes following the exclusion of deceased patients.

Within each cohort, changes in laboratory values from baseline to post-treatment were analyzed using paired *t*-test if the data met the normality assumption or the Wilcoxon signed-rank test otherwise. Analysis of covariance (ANCOVA) was then employed to analyze between-group differences in the changes of biomarkers from pre- to post-treatment, adjusting for baseline levels as covariates. A non-linear relationship between serum NfL levels and age was observed, so age-corrected Z-scores for serum NfL levels were obtained according to the previously published methods [29]. The cut-off value of anti-GM1 antibody positivity was defined as two SDs above the mean level in HCs [30, 31]. A two-tailed *P* value < 0.05 was considered statistically significant.

## Results

### Participants

**Fig. 1:**
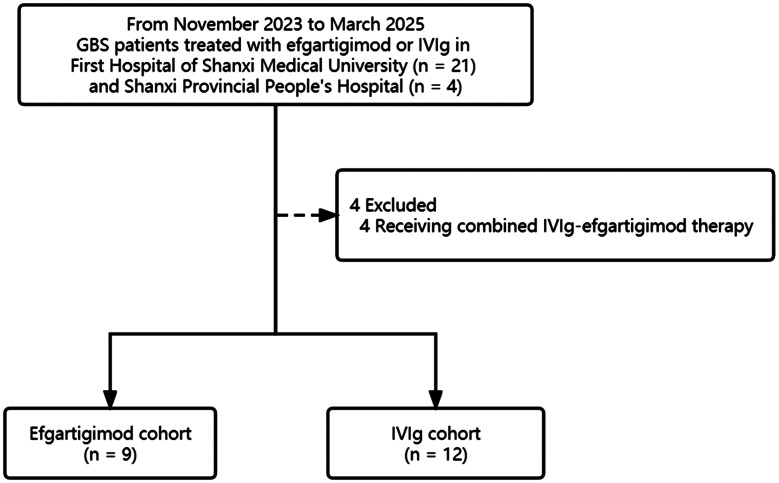
Flowchart of study participant selection

A total of 25 patients initially met the inclusion criteria and were enrolled in the study. During the follow-up period, 4 patients withdrew after receiving combination therapy with efgartigimod and IVIg. Ultimately, 21 patients were included, with 9 in the efgartigimod cohort and 12 in the IVIg cohort (Fig. 1). The demographic and clinical characteristics at baseline are summarized in Table 1. No significant differences were observed between the two cohorts in terms of age, sex, antecedent illness, days from onset to administration, disease severity (measured by the GBS-DS functional grade, INCAT disability score, I-RODS, grip strength, MRC sum score), GBS subtype, or serum albumin and IgG levels at baseline (*P* > 0.05).

**Table 1:** Baseline characteristics of patients with Guillain-Barré syndrome

	Efgartigimod (*n* = 9)	IVIg (*n* = 12)	*P* value
Age, years, mean ± SD	60.11 ± 14.46	61.50 ± 19.51	0.860
Sex, n, (%)			
Male	4 (44.44%)	6 (50.00%)	1.000
Female	5 (55.56%)	6 (50.00%)	
Antecedent illness, n, (%)			0.267
None	0 (0.00%)	3 (25.00%)	
Respiratory infection	6 (66.67%)	3 (25.00%)	
Diarrhoea	4 (44.44%)	5 (41.67%)	
Fever	2 (22.22%)	4 (33.33%)	
Days from onset to administration, mean ± SD	5.22 ± 2.86	4.92 ± 2.71	0.716
GBS-DS functional grade, n, (%)			1.000
3	0 (0.00%)	1 (8.33%)	
4	8 (88.89%)	9 (75.00%)	
5	1 (11.11%)	2 (16.67%)	
INCAT disability score, median (IQR)	9.00 (1.00)	9.00 (2.00)	0.602
I-RODS, median (IQR)	1.00 (6.00)	2.00 (3.50)	0.602
Grip strength, kg, (average of both sides), median (IQR)	0.69 (4.14)	3.07 (7.26)	0.422
Sum MRC score, mean ± SD	27.22 ± 11.79	34.83 ± 8.15	0.096
Subtype, n, (%)			0.475
AIDP	1 (11.11%)	0 (0.00%)	
AMAN	4 (44.44%)	8 (66.67%)	
AMSAN	3 (33.33%)	4 (33.33%)	
Unclassified	1 (11.1%)	0 (0.00%)	
Albumin, g/L, mean ± SD	40.37 ± 2.35	38.81 ± 3.52	0.265
IgG, g/L, mean ± SD	12.72 ± 4.91	16.13 ± 7.78	0.322

Abbreviation: IVIg, Intravenous immunoglobulin; GBS-DS, Guillain-Barré syndrome Disability Scale; INCAT, Inflammatory Neuropathy Cause and Treatment; I-RODS, Inflammatory Rasch-Built Overall Disability Scale; MRC, Medical Research Council; AIDP, acute inflammatory demyelinating polyneuropathy; AMAN, acute motor axonal neuropathy; AMSAN, acute motor-sensory axonal neuropathy; IgG, immunoglobulin G

### Outcomes

The proportion of patients achieving the primary outcome — defined as a GBS-DS functional grade ≤ 2 at 4 weeks post-treatment — was 3 of 9 (33.3%) in the efgartigimod cohort and 3 of 12 (25.0%) in the IVIg cohort (OR = 0.67, 95% CI [0.10, 4.48]; *P* = 1.000, Fisher’s exact test; Table 2). Regarding secondary outcomes (Table 2), the proportions of patients who improved ≥ 1 grade in GBS-DS from baseline at week 4 (OR = 0.40, 95% CI [0.07, 2.37]; *P* = 0.396) and week 24 (OR = 1.67, 95% CI [0.09, 31.87]; *P* = 1.000) were similar between the two cohorts. At week 24, 3 of 5 patients (60.00%) in the efgartigimod cohort and 6 of 9 patients (66.70%) in the IVIg cohort achieved a GBS-DS functional grade ≤ 2 (OR = 0.48, 95% CI [0.06, 3.63]; *P* = 0.637). And there was no difference between these two groups in the GBS-DS grade at week 4 (*P* = 0.893).

**Table 2:** Comparison of primary and secondary clinical outcomes between Efgartigimod and IVIg cohorts in the treatment of Guillain-Barré syndrome

	Efgartigimod	IVIg	*P* value
GBS-DS grade ≤ 2, (%)			
Week 4	3 / 9 (33.33%)	3 / 12 (25.00%)	1.000
Week 24	5 / 7 (71.43%)	6 / 11 (54.55%)	0.637
Improvement ≥ 1-grade in GBS-DS, (%)			
Week 4	5 / 9 (55.56%)	4 / 12 (33.33%)	0.396
Week 24	6 / 7 (85.71%)	10 / 11 (90.91%)	1.000
GBS-DS grade at week 4, (%)			0.893
1	0 / 9 (0.00%)	1 / 12 (8.33%)	
2	3 / 9 (33.33%)	2 / 12 (16.67%)	
3	2 / 9 (22.22%)	1 / 12 (8.33%)	
4	3 / 9 (33.33%)	4 / 12 (33.33%)	
5	1 / 9 (11.11%)	3 / 12 (25.00%)	
6	0 / 9 (0.00%)	1 / 12 (8.33%)	
Change in GBS-DS scores, median (IQR, n)			
Week 1	0.00 (0.00, *n* = 9)	0.00 (0.00, *n* = 12)	0.901
Week 2	−1.00 (1.00, *n* = 9)	0.00 (0.75, *n* = 12)	0.675
Week 4	−1.00 (2.00, *n* = 9)	0.00 (2.25, *n* = 12)	0.339
Week 8	−2.00 (4.50, *n* = 9)	−1.00 (3.00, *n* = 11)	0.789
Week 16	−1.00 (1.00, *n* = 8)	−1.00 (3.00, *n* = 11)	0.567
Week 24	−2.00 (1.00, *n* = 7)	−1.00 (2.00, *n* = 11)	0.846
Change in INCAT scores, median (IQR, n)			
Week 1	0.00 (0.50, *n* = 9)	0 (1.50, *n* = 12)	0.907
Week 2	−1.00 (1.00, *n* = 9)	0.00 (1.50, *n* = 12)	0.870
Week 4	−1.00 (3.00, *n* = 9)	0.00 (1.50, *n* = 12)	0.226
Week 8	−2.00 (4.50, *n* = 9)	−1.00 (3.00, *n* = 11)	0.350
Week 16	−3.00 (4.50, *n* = 8)	−4.00 (3.00, *n* = 11)	0.483
Week 24	−3.00 (5.00, *n* = 7)	−4.00 (3.00, *n* = 11)	0.477
Change in I-RODS scores, median (IQR, n)			
Week 1	0.00 (3.50, *n* = 9)	0.00 (2.50, *n* = 12)	0.967
Week 2	1.00 (7.50, *n* = 9)	0.00 (3.50, *n* = 12)	0.900
Week 4	3.00 (9.00, *n* = 9)	0.50 (10.00, *n* = 12)	0.941
Week 8	4.00 (15.50, *n* = 9)	2.00 (32.00, *n* = 11)	0.762
Week 16	8.00 (10.25, *n* = 8)	8.00 (30.00, *n* = 11)	0.800
Week 24	11.00 (13.00, *n* = 7)	13.00 (29.00, *n* = 11)	0.587
Change in grip strength, kg, median (IQR, n)			
Week 1	0.00 (4.39, *n* = 9)	−0.07 (1.75, *n* = 11)	0.232
Week 2	2.15 (6.51, *n* = 5)	0.00 (7.57, *n* = 7)	0.284
Week 4	1.48 (6.93, *n* = 5)	−0.43 (3.74, *n* = 8)	0.245
Week 8	2.91 (4.69, *n* = 4)	2.63 (6.01, *n* = 6)	0.813
Week 16	3.30 (9.17, *n* = 4)	3.23 (6.08, *n* = 4)	0.529
Week 24	3.09 (9.78, *n* = 4)	3.00 (10.23^*^, *n* = 3)	0.590
Change in MRC sum scores, median (IQR, n)			
Week 1	4.00 (10.00, *n* = 9)	−2.00 (4.00, *n* = 11)	0.016
Week 2	15.00 (16.50, *n* = 5)	−2.00 (22.00, *n* = 7)	0.002
Week 4	6.00 (22.50, *n* = 5)	−0.50 (21.00, *n* = 8)	0.008
Week 8	13.50 (18.25, *n* = 4)	3.00 (16.00, *n* = 6)	0.055
Week 16	6.50 (23.75, *n* = 4)	6.00 (26.50, *n* = 4)	0.107
Week 24	12.00 (12.75, *n* = 4)	4.00 (36.00^*^, *n* = 3)	0.008

^*^: represent total range

Abbreviation: GBS-DS, Guillain-Barré syndrome Disability Scale; IQR, interquartile range; INCAT, Inflammatory Neuropathy Cause and Treatment; I-RODS, Inflammatory Rasch-Built Overall Disability Scale; MRC, Medical Research Council

GLMM analyses revealed significant time-dependent improvements in GBS patients across all outcomes: GBS-DS (*F*_5, 108_ =17.88, *P* < 0.001), INCAT disability score (*F*_5, 108_ =26.33, *P* < 0.001), I-RODS (*F*_5, 108_ = 16.30, *P* < 0.001), grip strength (*F*_5, 58_ = 4.17, *P* = 0.003), and MRC sum score (*F*_5, 58_ = 10.16, *P* < 0.001). In contrast, treatment exposure had no significant main effects on GBS-DS (*F*_5, 108_ = 0.17, *P* = 0.680), INCAT disability score (*F*_5, 108_ = 0.36, *P* = 0.552), I-RODS (*F*_5, 108_ = 0.06, *P* = 0.806), or grip strength (*F*_5, 58_ = 0.94, *P* = 0.337), with the exception of MRC sum score (*F*_5, 58_ = 7.93, *P* = 0.007). No significant interaction effects between treatment exposure and time were detected for the GBS-DS, INCAT disability score, I-RODS, grip strength, or MRC sum score (*P* > 0.2). Post-hoc pairwise comparisons using the least significant difference (LSD) method revealed no significant between-group differences in the mean changes from baseline for GBS-DS, INCAT disability score, I-RODS, or grip strength at any follow-up timepoint between the two cohorts (*P* > 0.05; Table 2, Supplement Fig. 1). In contrast, the MRC sum score demonstrated significantly greater muscle strength improvements in the efgartigimod cohort than in the IVIg cohort at week 1 (Δ = 6, *P* = 0.016), week 2 (Δ = 17, *P* = 0.002), week 4 (Δ = 6.5, *P* = 0.008), and week 24 (Δ = 8, *P* = 0.008) post-treatment. Sensitivity analyses excluding a deceased patient produced qualitatively similar results (Supplement Table 1).

In the efgartigimod cohort, the safety profile included Grade 3 lung infections in 3 patients, Grade 2 or 3 elevated aminotransferases in 3 patients, Grade 2 myalgia in 1 patient, Grade 1 fever in 1 patient, and Grade 1 increased creatinine in 1 patient. In the IVIg cohort, 3 patients had Grade 3 lung infections, and 1 patient presented with Grade 2 elevated aminotransferases. An 87-year-old woman receiving IVIg therapy died of respiratory failure at 13 days post-treatment, following her refusal to undergo mechanical ventilation.

**Fig. 2:**
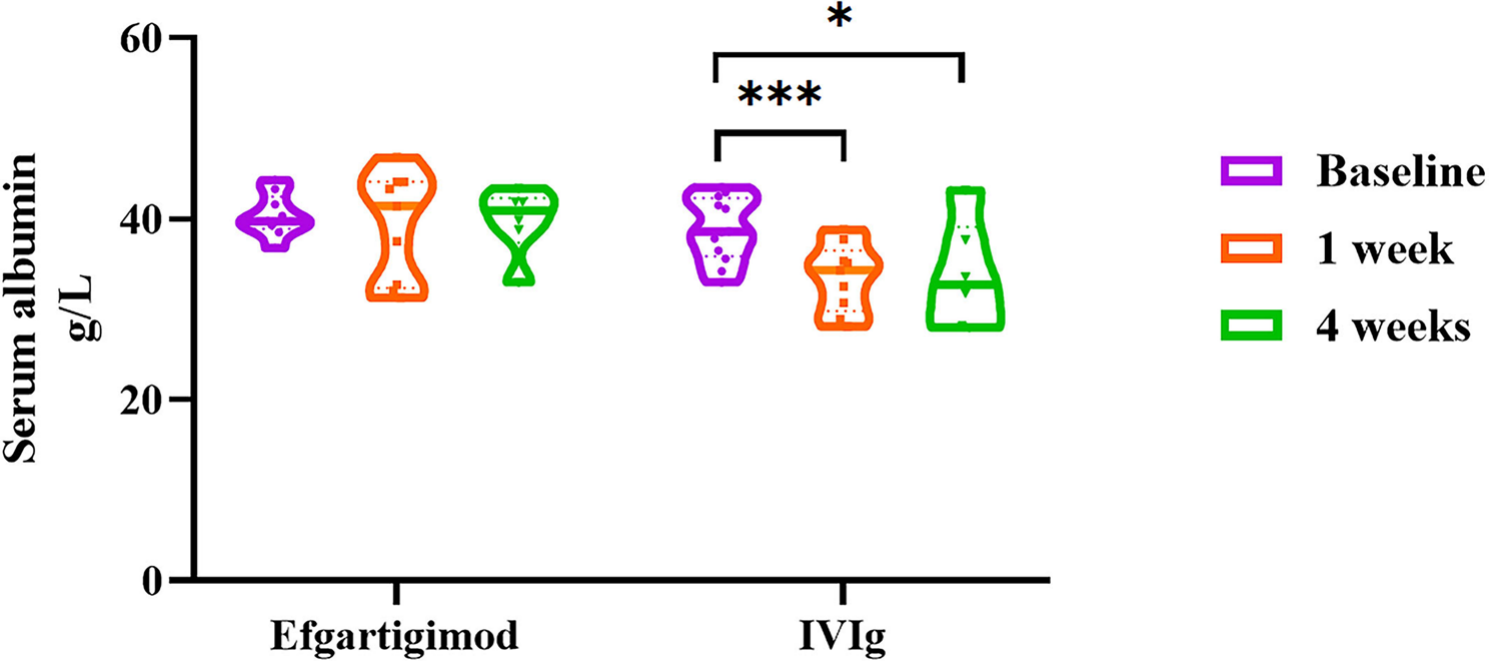
Albumin levels in GBS patients treated with efgartigimod and IVIg. Compared with baseline, the albumin levels in the efgartigimod cohort remained stable at both week 1 and week 4, whereas those in the IVIg cohort were significantly lower at week 1 and week 4 (paired *t*-test)

Pharmacodynamic analyses of serum IgG levels revealed a 65.53% (SD: 2.67%, *n* = 8) reduction in the efgartigimod cohort at 1 week post-treatment. Albumin levels remained stable in this cohort at both week 1 (mean difference: -1.13, 95% CI [-5.23, 2.98], *n* = 9; paired *t*-test, *P* = 0.548) and week 4 (mean difference: -0.39, 95% CI [-3.20, 2.43], *n* = 6; *P* = 0.748) compared with baseline. Conversely, the IVIg cohort exhibited significant reductions in albumin levels at week 1 (mean difference: -4.97, 95% CI [-7.15, -2.79], *n* = 10; *P* < 0.001) and week 4 (mean difference: -6.27, 95% CI [-11.44, -1.09], *n* = 6; *P* = 0.026) (Fig. 2).

### NfL in GBS

Baseline serum samples were analyzed from 14 GBS patients (efgartigimod: *n* = 7; IVIg: *n* = 7) and 1-week post-treatment samples from 11 patients (efgartigimod: *n* = 5; IVIg: *n* = 6), alongside samples from 13 HCs.

**Fig. 3:**
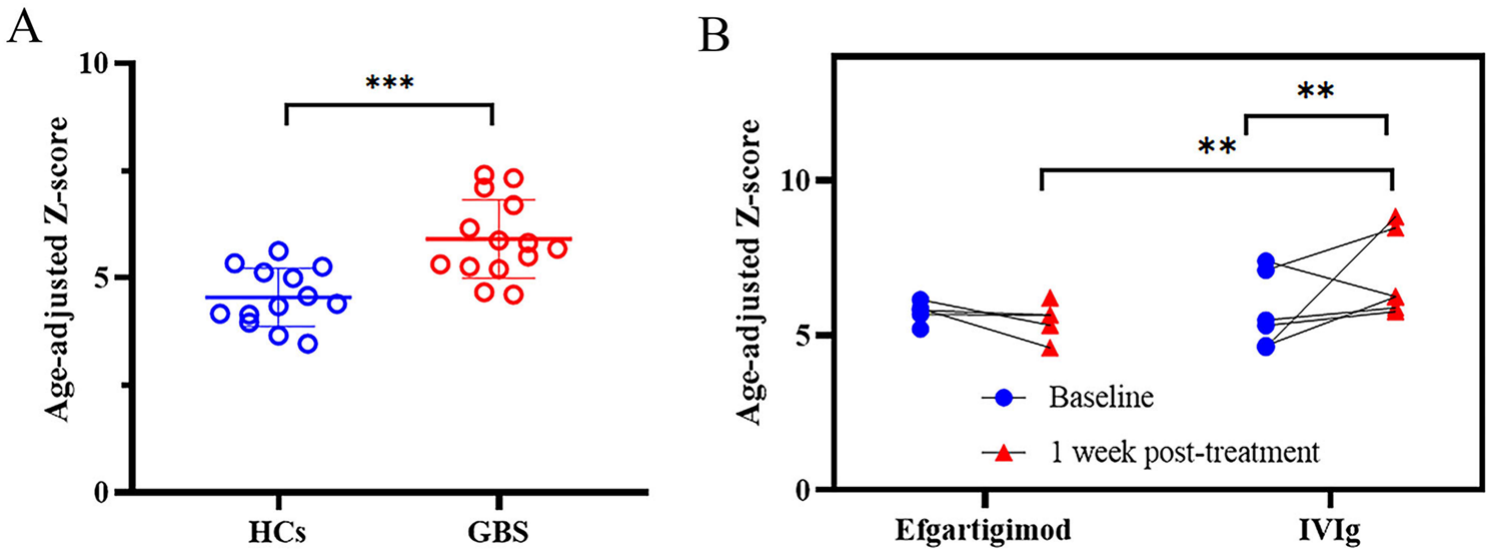
Age-adjusted Z-scores in GBS patients treated with efgartigimod and IVIg. (**A**) The pre-treatment age-adjusted Z-scores in GBS patients were significantly higher than those in HCs (independent-samples *t*-test). (**B**) At 1 week post-treatment of age-adjusted Z-scores, the IVIg cohort showed a significant increase in compared to baseline, while the efgartigimod cohort remained stable (paired *t*-test); adjusting for baseline values revealed substantially higher in the IVIg cohort compared to the efgartigimod group (ANCOVA)

Pre-treatment age-adjusted Z-scores were significantly elevated in GBS patients compared to HCs (mean difference: 1.36, 95% CI [0.71, 2.00]; GBS: *n* = 14; HCs: *n* = 13; independent-samples *t*-test, *P* < 0.001; Fig. 3). This elevation persisted when analyzing raw serum NfL (mean difference: 70.64 pg/mL, 95% CI [54.22, 87.06]; *P* < 0.001; Supplement Fig. 2A). No significant differences were observed between the efgartigimod and IVIg cohorts in either age-adjusted Z-scores (mean difference: 0.00, 95% CI [-1.12, 1.11]; efgartigimod: *n* = 7; IVIg: *n* = 7; *P* = 0.996) or raw serum NfL levels (mean difference: -16.70 pg/mL, 95% CI [-44.86, 11.46]; *P* = 0.221).

At 1 week post-treatment, the IVIg cohort exhibited a significant increase in age-adjusted Z-scores compared with the baseline (mean difference: 1.15, 95% CI [0.80, 1.50]; *n* = 6; paired *t*-test, *P* = 0.004), whereas the efgartigimod cohort maintained stable Z-scores (mean difference: -0.26, 95% CI [-0.56, 0.044]; *n* = 5; *P* = 0.077) (Fig. 3B). Raw serum NfL levels mirrored this pattern (Supplement Fig. 2B).

In patients with paired baseline and 1-week data, age-adjusted Z scores were not significantly different at baseline between the efgartigimod and IVIg cohorts (mean difference: 0.02, 95% CI [-1.25, 1.29]; efgartigimod: *n* = 5; IVIg: *n* = 6; independent-samples *t*-test, *P* = 0.974). However, raw serum NfL levels were significantly lower in the IVIg cohort at baseline (mean difference: -34.37 pg/mL, 95% CI [-58.17, -10.58]; *P* = 0.010). ANCOVA adjusted for baseline values revealed substantially higher age-adjusted Z-scores in the IVIg cohort compared to the efgartigimod cohort at week 1 (adjusted mean difference: 1.40, 95% CI [1.00, 1.80]; *P* < 0.001; Fig. 3B). This pattern was consistent with the raw serum NfL data (adjusted mean difference: 135.38 pg/mL, 95% CI [27.26, 243.50]; *P* = 0.020; Supplement Fig. 2B).

### Anti-GM1 antibody in GBS

**Fig. 4:**
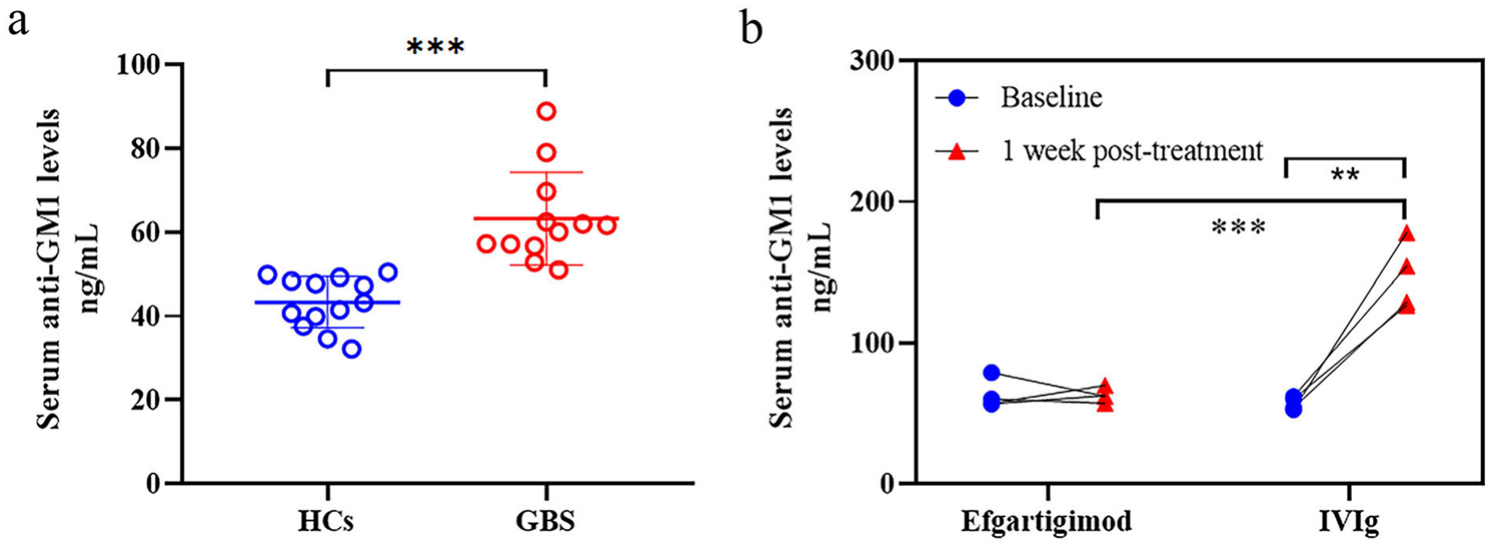
IgG antibodies against GM1 in GBS patients treated with efgartigimod and IVIg. (**A**) The pre-treatment anti-GM1 levels were significantly higher in GBS patients than in HCs (Mann‒Whitney *U* test). (**B**) At 1 week post-treatment, the IVIg cohort showed a significant increase in anti-GM1 levels compared with the baseline (paired *t*-test), whereas the efgartigimod cohort remained stable (Wilcoxon signed-rank test); adjusting for baseline values revealed substantially higher in the IVIg cohort than in the efgartigimod group (ANCOVA)

On the basis of the distribution of anti-GM1 levels in 13 HCs (43.27 ± 6.07 ng/mL), the diagnostic cutoff value for antibody seropositivity was mathematically determined as 55.42 ng/mL (mean + 2 SD). Given the current lack of established specific antibody biomarkers for AIDP [32], anti-GM1 antibody testing was selectively conducted in patients presenting with AMAN and AMSAN subtypes. Baseline serum samples were obtained from 12 GBS patients with AMAN/AMSAN subtypes (efgartigimod: *n* = 5; IVIg: *n* = 7). Comparative analysis revealed significantly elevated serum anti-GM1 antibody levels in GBS patients compared with HCs (median difference: 17.60 ng/mL; GBS: *n* = 12; HCs: *n* = 13; Mann‒Whitney *U* test, *P* < 0.001; Fig. 4A). Among the 12 patients, 10 were positive for anti-GM1 (efgartigimod: *n* = 5; IVIg: *n* = 5). No significant differences were detected between the efgartigimod and IVIg cohorts in anti-GM1 levels among patients who tested positive for anti-GM1 (median difference: 2.36 ng/mL; efgartigimod: *n* = 5; IVIg: *n* = 5; Mann‒Whitney *U* test, *P* = 0.310).

At 1-week post-treatment, serial serum samples were obtained from 8 of the 10 anti-GM1 seropositive patients (efgartigimod: *n* = 4; IVIg: *n* = 4). Longitudinal analysis revealed distinct pharmacodynamic profiles: the efgartigimod cohort showed no significant change in anti-GM1 levels from baseline (median of differences: -5.39 ng/mL; Wilcoxon signed-rank test, *P* = 0.250), whereas the IVIg cohort exhibited a marked elevation in antibody concentrations (mean of differences: 84.12 ng/mL, 95% CI [52.21, 116.0]; paired *t*-test, *P* = 0.004) (Fig. 4B).

In patients with paired baseline and 1-week data, anti-GM1 antibody levels showed comparable baseline values between the efgartigimod and IVIg treatment cohorts (median difference: 3.55 ng/mL; efgartigimod: *n* = 4; IVIg: *n* = 4; Mann‒Whitney *U* test, *P* = 0.688). However, ANCOVA adjusted for baseline measurements revealed a clinically significant difference at week 1 post-treatment, with the IVIg cohort demonstrating substantially elevated anti-GM1 levels compared with the efgartigimod cohort (adjusted mean difference: 90.19 ng/mL, 95% CI [-120.87, 59.52]; *P* < 0.001; Fig. 4B).

## Discussion

This dual-center retrospective cohort study did not demonstrate statistically significant differences between efgartigimod and IVIg in the primary outcome of independent ambulation proportion at 4 weeks post-treatment in GBS management (*P* = 1.000), with both regimens exhibiting favorable safety profiles. However, analyses revealed favorable trends with efgartigimod across multiple other measures: greater improvement in MRC sum score (*P* = 0.007), and a more favorable trajectory of NfL and anti-GM1 levels (*P* < 0.001). These findings suggest that efgartigimod has potential as a clinically equivalent alternative to IVIg. In addition, this study allows us to estimate the effect size of efgartigimod and its SD for future trials.

Currently, two therapies are established for GBS: IVIg and PLEX [33]. However, these treatments do not fully address clinical needs because of variable efficacy, safety concerns, and limited accessibility [9, 10, 34]. Although developing novel therapies is imperative, designing randomized controlled trials (RCTs) poses significant ethical and statistical challenges. The use of placebo controls would be ethically unacceptable, as withholding IVIg or PLEX—proven life-saving interventions—violates the Declaration of Helsinki’s mandate to prioritize patient welfare. However, both active-controlled superiority trials and non-inferiority trials typically necessitate large participant cohorts to maintain sufficient statistical power—a particular barrier for rare diseases such as GBS [35]. One ongoing registered trial (NCT05701189) evaluating efgartigimod in patients with GBS was prospectively designed with a small enrollment target of 30 subjects. As a result, the development of optimized clinical endpoints is methodologically imperative to achieve maximal equilibrium between sample size requirements and statistical power in GBS trial design.

The GBS-DS was selected as the primary outcome in this study, aligning with most RCTs [36, 37]. However, this 7-point ordinal scale (range 0–6) demonstrates limited sensitivity in detecting marginal clinical improvements, as it primarily focuses on ambulation and respiration, but not on arms or other functions [2]. In contrast, the MRC sum score provides a more comprehensive quantification of both upper and lower limb muscle strength, potentially offering greater sensitivity. A previous RCT investigating the efficacy of IVIg and PLEX in GBS designated both the MRC sum score and the GBS-DS as primary endpoints, which revealed a significant difference in the time course of recovery between treatment groups when using the MRC sum score as the outcome measure, whereas no between-group difference was observed when the GBS-DS was utilized [38]. Therefore, adopting the change in MRC sum score as the primary endpoint may represent a methodologically sound approach in future GBS clinical investigations.

Elevated serum NfL levels are significantly correlated with the clinical features of GBS, including disease severity, axonal degeneration, and unfavorable prognosis [39–42]. These findings suggest that the serum NfL level may serve as an intermediate endpoint in clinical trials to objectively assess therapeutic efficacy [39]. In this study, we evaluated changes in serum NfL levels at 1 week post-treatment as an exploratory outcome. A significant increase in NfL levels was observed at 1 week after IVIg therapy (*P* = 0.004), which is consistent with prior evidence of peak concentrations around day 20 post-treatment [39]. In contrast, patients treated with efgartigimod presented NfL levels comparable to those at baseline at the 1-week timepoint (*P* = 0.077), which were significantly lower than those in the IVIg cohort (*P* < 0.001). The results align with the observed significant between-group differences in MRC sum score improvements at the same post-treatment interval (*P* = 0.016), and suggest a more pronounced attenuation of axonal injury by the efgartigimod regimen.

Anti-GM1 antibodies are established pathogenic drivers in AMAN and AMSAN [43], accounting for 90.5% of the enrolled patients in this cohort. Among patients with a high anti-GM1 IgG titer at baseline, a slow titer decline was associated with poor outcomes at 4 weeks and 6 months [44]. Therefore, we defined the change in GM1 antibody levels from baseline to week 1 as the other exploratory outcome in the present study, and observed parallel trajectories between GM1 antibody levels and NfL concentrations across both the efgartigimod and IVIg cohorts. Anti-GM1 antibody levels showed variable and individualized responses following IVIg treatment, and an increase in GM1 IgG antibody titers can also occur in GBS patients who had not received IVIg treatment [45], which suggested that IVIg itself does not lead to an increase in GM1 antibody titers. Rayomand Press et al. considered the occurrence of a peak phenomenon for anti-GM1 antibodies after IVIg administration appears to be independent of the infused IVIg itself [45]. We propose that this variability may stem from differences in individual immune status. These findings suggest that efgartigimod is more effective than IVIg in reducing GM1 IgG antibody titers in GBS patients.

The FcRn rescues albumin and IgG from degradation following endocytosis and thereby extends the half-life of these plasma proteins [46]. IVIg can accelerate the catabolism of IgG antibodies by saturating the FcRn highly expressed on vascular endothelial cells [47]. Our findings, in alignment with prior research, demonstrate that IVIg significantly reduced albumin levels [48]. This albumin-lowering effect may be explained by saturation of FcRn-mediated albumin recycling, which potentially accelerates its catabolism [48]. Certain FcRn-specific antibodies, such as nipocalimab, can also result in reductions in the levels of serum albumin [49, 50]. In fact, sites for IgG and albumin on FcRn encompass distinct residues and do not overlap, and efgartigimod, owing to its unique molecular design, results in no clinically significant alterations in patient albumin levels [50].

This study has several limitations. First, the sample size was small and led to an imbalance in GBS subtypes; notably, no AIDP cases were included in the IVIg group, which may limit the generalizability of our findings. The limited sample size reduced the statistical power to detect between-cohort differences in treatment efficacy. Second, the retrospective design resulted in missing data points for certain parameters at specific time intervals, potentially compromising the validity of the findings. Third, the applicability to GBS of the efgartigimod dosing regimen remains uncertain. Given the acute and monophasic nature of GBS, where the antibody titers peak within the first week, we hypothesized that two doses of efgartigimod could provide sufficient pharmacodynamic coverage during the critical disease window. Future studies are required to evaluate whether extended schedules or higher cumulative doses may further improve outcomes and thereby better clarify the therapeutic potential of efgartigimod in GBS.

## Conclusion

Compared with IVIg, efgartigimod demonstrated significantly greater improvements in MRC sum scores and more favorable trajectories in NfL levels and anti-GM1 antibody titers in patients with GBS based on our limited sample size. Although the between-cohort difference did not reach statistical significance for the primary outcome, the consistent benefits observed across multiple secondary and exploratory measures suggest that efgartigimod may serve as a promising alternative to IVIg in GBS management.

## Supplementary Information

Below is the link to the electronic supplementary material.

Supplementary Material 1: Supplement Fig. 1 Comparison of secondary clinical outcomes between efgartigimod and IVIg cohorts in the treatment of GBS (GLMM). (A-C) There were no significant between-group differences in the mean changes from baseline for GBS-DS, INCAT disability score and I-RODS at any follow-up timepoint between the two cohorts. (D) Change in grip strength at week 1 showed no significant differences between the two cohorts. (E) Change in MRC sum score at week 1 demonstrated a statistically significant difference between the two cohorts. Data for grip strength and MRC sum score at the week 1 are presented due to the availability of more complete data at this time

Supplementary Material 2: Supplement Fig. 2 Raw serum NfL levels in GBS patients treated with efgartigimod and IVIg. (A) The pre-treatment raw serum NfL levels in GBS patients were significantly higher than HCs (independent-samples t-test). (B) At 1 week post-treatment, the IVIg cohort showed a significant increase in serum NfL levels compared with those at baseline, whereas the efgartigimod cohort remained stable (paired t test); adjusting for baseline values revealed substantially higher in the IVIg cohort compared to the efgartigimod group (ANCOVA)

Supplementary Material 3

## Data Availability

The datasets used and/or analyzed during the current study are available from the corresponding author upon reasonable request.

## Abbreviations

IVIg: Intravenous immunoglobulin
GBS-DS: Guillain-Barré syndrome Disability Scale
INCAT: Inflammatory Neuropathy Cause and Treatment
I-RODS: Inflammatory Rasch-Built Overall Disability Scale
MRC: Medical Research Council
AIDP: Acute inflammatory demyelinating polyneuropathy
AMAN: Acute motor axonal neuropathy
AMSAN: Acute motor-sensory axonal neuropathy
IgG: Immunoglobulin G
