# Supplementary figures and images for: Comparison of intravenous efgartigimod and intravenous immunoglobulin in patients with Guillain–Barré syndrome
Source: Orphanet J Rare Dis. 2025 Oct 21;20:529. doi: 10.1186/s13023-025-04060-0 (PMC12542069; doi:10.1186/s13023-025-04060-0)

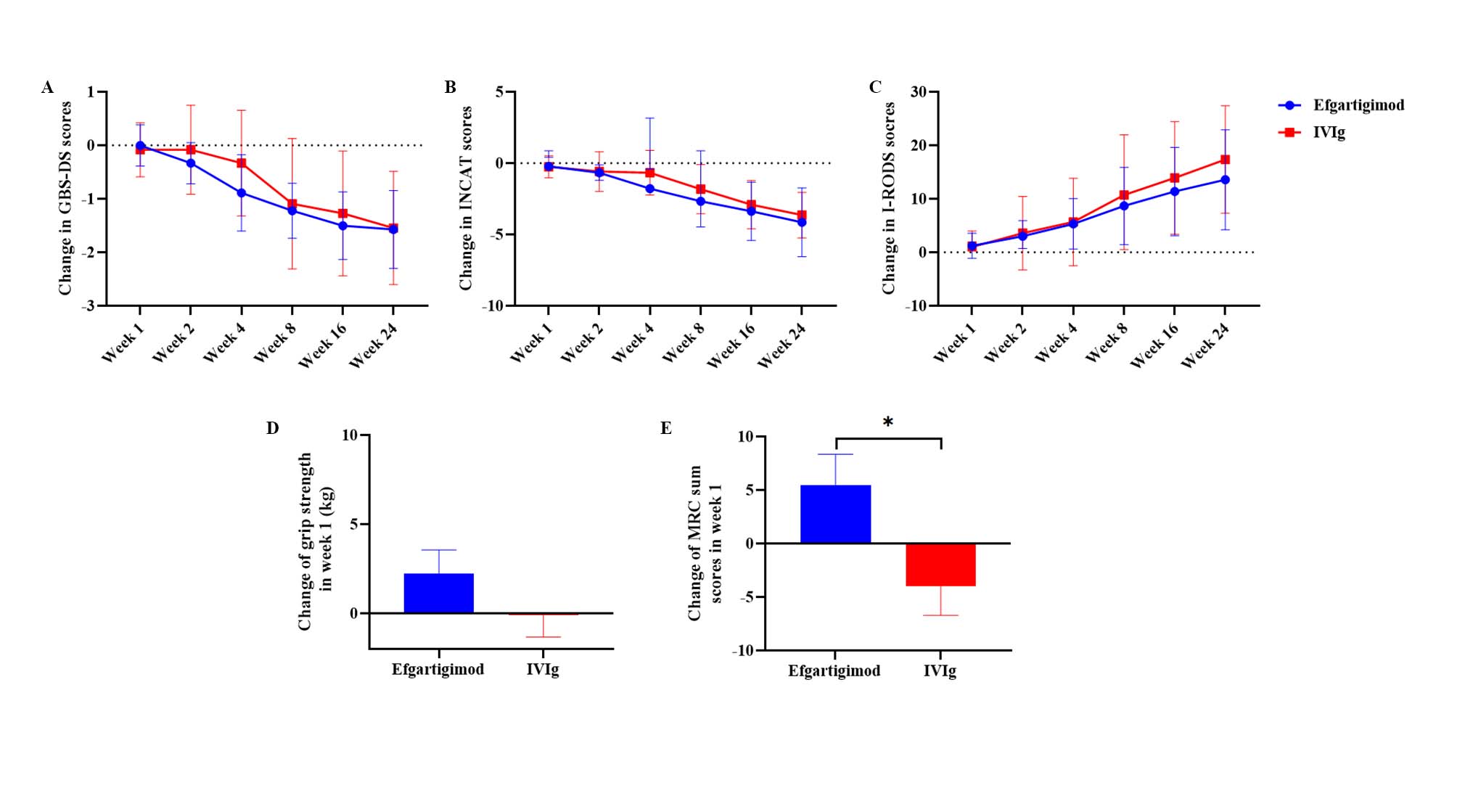

Supplement: Supplementary file 1 — Supplementary Material 1: Supplement Fig. 1 Comparison of secondary clinical outcomes between efgartigimod and IVIg cohorts in the treatment of GBS (GLMM). (A-C) There were no significant between-group differences in the mean changes from baseline for GBS-DS, INCAT disability score and I-RODS at any follow-up timepoint between the two cohorts. (D) Change in grip strength at week 1 showed no significant differences between the two cohorts. (E) Change in MRC sum score at week 1 demonstrated a statistically significant difference between the two cohorts. Data for grip strength and MRC sum score at the week 1 are presented due to the availability of more complete data at this time [file 13023_2025_4060_MOESM1_ESM.jpg]

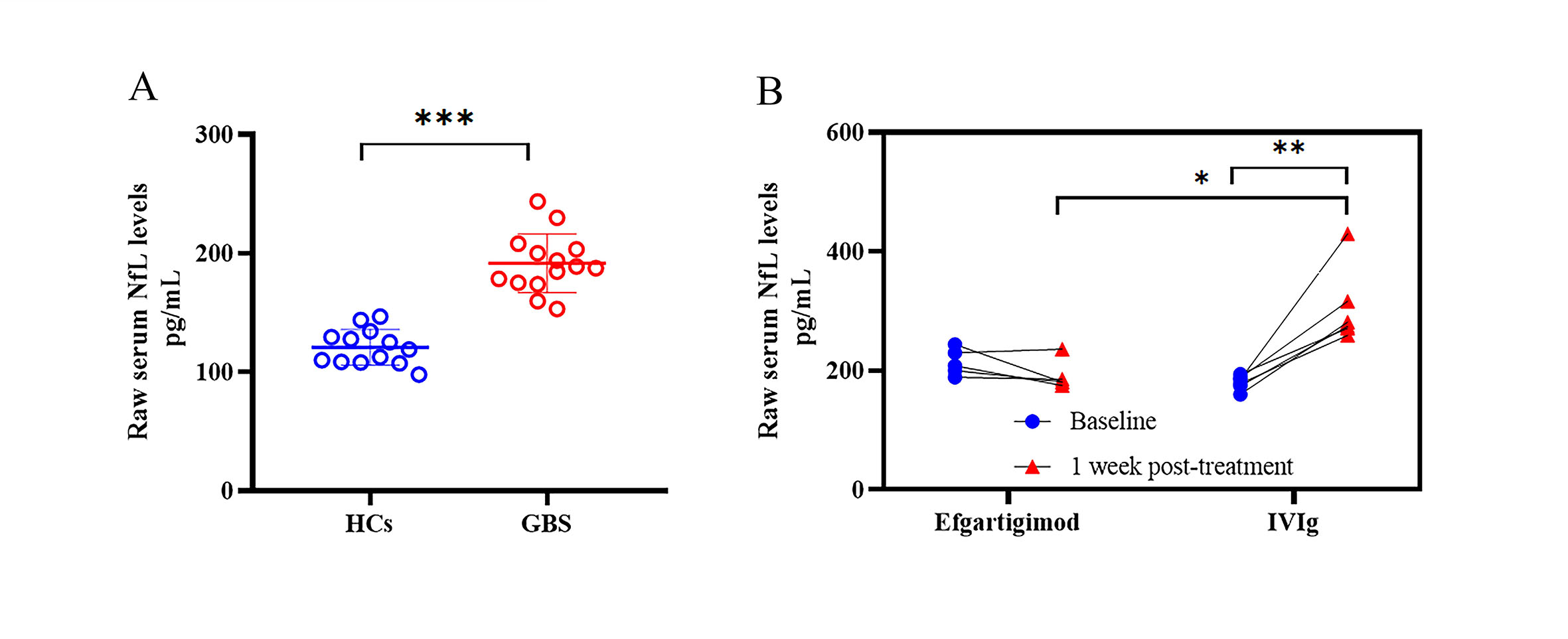

Supplement: Supplementary file 2 — Supplementary Material 2: Supplement Fig. 2 Raw serum NfL levels in GBS patients treated with efgartigimod and IVIg. (A) The pre-treatment raw serum NfL levels in GBS patients were significantly higher than HCs (independent-samples t-test). (B) At 1 week post-treatment, the IVIg cohort showed a significant increase in serum NfL levels compared with those at baseline, whereas the efgartigimod cohort remained stable (paired t test); adjusting for baseline values revealed substantially higher in the IVIg cohort compared to the efgartigimod group (ANCOVA) [file 13023_2025_4060_MOESM2_ESM.jpg]
