# Supplementary material for: Comparison of intravenous efgartigimod and intravenous immunoglobulin in patients with Guillain–Barré syndrome
Source: Orphanet J Rare Dis. 2025 Oct 21;20:529. doi: 10.1186/s13023-025-04060-0 (PMC12542069; doi:10.1186/s13023-025-04060-0)
Supplement: Supplementary file 3 — Supplementary Material 3 [file 13023_2025_4060_MOESM3_ESM.docx]

**Supplement table 1.** Sensitivity analysis, conducted by excluding a deceased patient, compared the primary and secondary clinical outcomes between the efgartigimod and IVIg cohorts in the treatment of Guillain-Barré syndrome

|  | Efgartigimod | IVIg | *P* value |
| --- | --- | --- | --- |
| GBS-DS grade ≤ 2, (%) | | | |
| Week 4 | 3 / 9 (33.33%) | 3 / 11 (27.27%) | 1.000 |
| Week 24 | 3 / 5 (60.00%) | 6 / 8 (75.00%) | 1.000 |
| Improvement ≥ 1-grade in GBS-DS, (%) | | | |
| Week 4 | 5 / 9 (55.56%) | 4 / 11 (36.36%) | 0.653 |
| Week 24 | 4 / 5 (80.00%) | 8 / 8 (100.00%) | 0.385 |
| GBS-DS grade at week 4, (%) |  |  | 0.850 |
| 1 | 0 / 9 (0.00%) | 1 / 11 (9.09%) |  |
| 2 | 3 / 9 (33.33%) | 2 / 11 (18.18%) |  |
| 3 | 2 / 9 (22.22%) | 1 / 11 (9.09%) |  |
| 4 | 3 / 9 (33.33%) | 4 / 11 (36.36%) |  |
| 5 | 1 / 9 (11.11%) | 3 / 11 (27.2%) |  |
| Change in GBS-DS scores, median (IQR, n) | | | |
| Week 1 | 0.00 (0.00, n = 9) | 0.00 (0.00, n = 11) | 0.855 |
| Week 2 | -1.00 (1.00, n = 9) | 0.00 (0.00, n = 11) | 0.901 |
| Week 4 | -1.00 (2.00, n = 9) | 0.00 (2.00, n = 11) | 0.494 |
| Week 8 | -2.00 (4.50, n = 9) | -1.00 (3.00, n = 10) | 0.745 |
| Week 16 | -1.00 (1.00, n = 8 ) | -1.50 (3.00, n = 10) | 0.956 |
| Week 24 | -2.00 (1.50, n = 5) | -2.00 (3.00, n = 8) | 0.514 |
| Change in INCAT scores, median (IQR, n) | | | |
| Week 1 | 0.00 (0.50, n = 9) | 0.00 (2.00, n = 11) | 0.876 |
| Week 2 | -1.00 (1.00, n = 9) | 0.00 (1.00, n = 11) | 0.834 |
| Week 4 | -1.00 (3.00, n = 9) | 0.00 (2.00, n = 11) | 0.244 |
| Week 8 | -2.00 (4.50, n = 9) | -1.00 (3.00, n = 10) | 0.465 |
| Week 16 | -3.00 (4.50, n = 8 ) | -4.00 (3.00, n = 10) | 0.704 |
| Week 24 | -3.00 (4.50, n = 5) | -5.00 (2.00, n = 8) | 0.855 |
| Change in I-RODS socres, median (IQR, n) | | | |
| Week 1 | 0.00 (3.50, n = 9) | 0.00 (3.00, n = 11) | 0.994 |
| Week 2 | 1.00 (7.50, n = 9) | 0.00 (4.00, n = 11) | 0.833 |
| Week 4 | 3.00 (9.00, n = 9) | 1.00 (12.00, n = 11) | 0.837 |
| Week 8 | 4.00 (15.50, n = 9) | 2.50 (32.00, n = 10) | 0.620 |
| Week 16 | 8.00 (10.25, n = 8) | 8.00 (30.00, n = 10) | 0.613 |
| Week 24 | 9.00 (17.00, n = 5) | 17.00 (25.00, n = 8) | 0.196 |
| Change in grip strength, kg, median (IQR, n) | | | |
| Week 1 | 0.00 (4.39, n = 9) | -0.07 (1.75, n = 11) | 0.259 |
| Week 2 | 2.15 (6.51, n = 5) | -1.50 (7.63, n = 6) | 0.282 |
| Week 4 | 1.48 (6.93, n = 5) | -0.85 (3.88, n = 7) | 0.275 |
| Week 8 | 2.91 (4.69, n = 4) | 3.37 (6.62, n = 5) | 0.998 |
| Week 16 | 3.30 (9.17, n = 4 ) | 5.90 (5.78^*^, n = 3) | 0.781 |
| Week 24 | 3.09 (9.78, n = 4) | 6.62 (7.23^*^, n = 2) | 0.935 |
| Change of MRC sum socre, median (IQR, n) | | | |
| Week 1 | 4.00 (10.00, n = 9) | -2.00 (4.00, n = 11) | 0.029 |
| Week 2 | 15.00 (16.50, n = 5) | -2.00 (13.00, n = 6) | 0.003 |
| Week 4 | 6.00 (22.50, n = 5) | 0.00 (8.00, n = 7) | 0.020 |
| Week 8 | 13.50 (18.25, n = 4) | 4.00 (10.00, n = 5) | 0.137 |
| Week 16 | 6.50 (23.75, n = 4 ) | 8.00 (6.00^*^, n = 3) | 0.305 |
| Week 24 | 12.00 (12.75, n = 4) | 8.00 (8.00^*^, n = 2) | 0.038 |

^*^: represent total range.

Abbreviation: GBS-DS, Guillain-Barré syndrome Disability Scale; IQR, interquartile range; INCAT, Inflammatory Neuropathy Cause and Treatment; I-RODS, Inflammatory Rasch-Built Overall Disability Scale; MRC, Medical Research Council.
